# Diagnostic delays in infective discitis – an unresolved problem

**DOI:** 10.1038/s41394-025-00706-0

**Published:** 2025-04-18

**Authors:** Lisa Grandidge, Tokunbo Ogunleye, Michael Thackray, Pradeep Thumbikat

**Affiliations:** 1 Consultant in Spinal Injuries. Princess Royal Spinal Injuries Unit, Northern General Hospital, Sheffield, UK; 2 Senior House Officer, Sheffield Teaching Hospitals, Sheffield, UK

**Keywords:** Physical examination, Risk factors, Disability

## Abstract

**Study Design:**

A retrospective study.

**Objectives:**

Neurological deficits occur in 1/3 of spinal infection cases. Non-specific symptoms can lead to delays in diagnosis. This study reviews outcomes and the timeliness of diagnosis in patients with spinal infection who sustained subsequent spinal cord impairment.

**Setting:**

All 40 patients admitted to The Princess Royal Spinal Injuries Centre, Sheffield, UK, between 2016–2018 for rehabilitation for spinal cord impairment secondary to spinal infection.

**Results:**

The average age was 58.6 years (31–85; SD 13.1) with 24 (60%) being male. 36 (90%) had native infections and 3 (7.5%) were acquired post-operatively (1 (2.5%) unknown). 7 patients had been intravenous drug users (17.5%). 50% (20) had previously sought medical attention for the same symptoms. There were long intervals to suspected diagnosis and to MRI (range 0–30 days). 15 (37.5%) patients deteriorated neurologically following admission to hospital; 8 were taking antibiotics at the time of deterioration. None of the patients had normal C-reactive protein levels at presentation. 7 (17.5%) sustained complete spinal cord impairment. 27 (67.5%) were discharged as wheelchair users.

**Conclusions:**

Whilst the majority of patients responded to rehabilitation interventions, they were left with residual life changing disabilities. Among those requiring rehabilitation, delays in diagnosis appear to frequently occur pre-hospital. This could be attributed to a low index of suspicion in the community. Some deteriorated neurologically despite antibiotics. Close neurological monitoring of those suspected or confirmed to have a diagnosis of spinal infection is appropriate. There should be a low threshold for the use of inflammatory markers when investigating back pain.

## Introduction

This study outlines the clinical presentation and management of patients with spinal infections admitted for rehabilitation over a 3-year period; we review the initial acute management and the outcomes of long-term rehabilitation, addressing the impact in terms of morbidity and mortality. We also review factors leading to diagnostic delays.

Spinal infections are relatively rare, with an incidence between 1:100,000 and 1:250,000 in developed countries [1]. Risk factors include older age, intravenous drug use, diabetes, HIV, malnutrition, chronic renal or liver disease, steroid use and previous spinal surgery or interventions. Incidence is increasing, potentially due to aging population, higher numbers of intravenous drug users, widespread use of immunosuppressive drugs and increasing prevalence of diabetes as well as improved diagnostic sensitivity [2, 3]. Staphylococcus aureus is the most commonly implicated pathogen [4].

The non-specific nature of the presentation frequently leads to delayed diagnosis, increasing consequential morbidity. The documented “classic triad” of fever, back pain and neurologic deficit is not a sensitive marker for spinal infection [5]. Prompt recognition and treatment is critical to managing spinal infection and thus minimising neurological sequelae. Reported factors contributing to diagnostic delay include a low index of suspicion and delays to investigations [5]. One third of those who develop infective discitis have neurological deficits [6].

The aim of this study is to identify delays in diagnosis and outcomes in patients with spinal infection who sustained subsequent spinal cord impairment.

## Materials and methods

The Princess Royal Spinal Injuries Centre covers a wide geographical area including 5 teaching hospitals and a catchment population of 10 million people. All 40 patients admitted to The Princess Royal Spinal Injuries Centre, Sheffield, UK, between 2016 and 2018 for rehabilitation for spinal cord injury secondary to spinal infection were included in this study. Patients were identified via coding, the National Spinal Cord Injury database, and the Sheffield Teaching Hospitals Spinal Infection multi-disciplinary team records. This was a retrospective study of clinical presentation and outcomes.

A proforma was created, piloted on 4 patients and then amended. The project was registered with the local clinical effectiveness unit. Electronic and paper records and electronic databases were reviewed. We did not have access to pre-transfer electronic records for some patients who were transferred from other hospitals. We extracted the following data: demographics, initial hospital, date of admission, symptoms, time to suspected diagnosis, time to MRI, investigations performed and results including microbiology, time to antibiotics, antibiotic type and course length, surgery date and type, neurological weakness at presentation and if there was deterioration, management of bladder and bowels at discharge and mobility, activities of daily living (ADLS) and transfers at discharge. The neurological status was recorded as per the American Spinal Injury Association score (ASIA or AIS). ASIA is synonymous with The International Standards for Neurological Classification of Spinal Cord Injury (ISNCSCI). Status (alive/deceased) at one year was also recorded. Descriptive statistics were used. A p-value of <0.05 was used for statistical significance.

There were two patients diagnosed with Mycobacterium Tuberculosis infections. These patients were included in the data analysis (unless stated otherwise); however, we acknowledge the difference in the presentation and management of patients with such diagnoses.

## Results

Patients had been transferred to the Princess Royal Spinal Injuries Centre from 17 different hospitals. The number of individuals with spinal infection who did not require specialist rehabilitation or transfer is unknown.

The mean age at admission was 58.6 years (range 31–85, median 57). 24 (60%) were male and 16 (40%) were female. 36 (90%) had native infections while 3 were post-operative (1 unknown). 7 patients were intravenous drug users (17.5%).

There were risk factors present for 18 patients. 22 patients (55%) had no risk factors (see Table 1).

**Table 1:** The risk factors for spinal infection present.

Risk Factor	Number (n = 18)
Diabetes	10
Hepatitis C	2
Hepatitis C and Alcohol excess	1
Rheumatoid arthritis and end stage renal failure	1
Autoimmune hepatitis and pancytopenia	1
Long-term steroid therapy	1
Alcohol excess	1
HIV infection	1

The mean length of stay at the spinal injuries centre was 102 days (range 21–526 days The mean length of overall hospital admission, was 180 days (median 162, range 56–617 days). The patient admitted for the highest number of days was a high-level ventilated patient with tetraplegia.

The median duration of symptoms prior to presentation was 12 days (range 1–180 days; SD 35.1). The patient with symptoms of 180 days had a longstanding history of back pain and was treated for tuberculosis in the intervening period. The median number of days between admission and the diagnosis of discitis being suspected was 1 day, with a range of 0 to 30 days (SD 6.5, mean 3.6 days).

Half of patients (20; 50%) had previously sought medical attention for the same symptoms before presentation to the admitting medical facility. 3 developed spinal infection following a spinal procedure. 33 (82.5%) patients had neck or back pain at presentation (see Table 2). 7 (17.5%) patients had red flag signs of sepsis and 13 (32.5%) patients were pyrexial at presentation. 31 patients (77.5%) had a neurological deficit at the time of the suspected diagnosis (1 unknown). 14 (35%) patients required an admission to High Dependency or Intensive Care Units (1 unknown).

**Table 2:** An overview of presenting features.

Feature	No.	%
Neck or back pain at presentation	33	82.5%
Pyrexial at presentation	13	32.5%
Signs of sepsis at presentation	7	17.5%
Neurological deficit at the time of suspected diagnosis	31	77.5%
Number of patients who sought medical attention on at least one occasion prior to index presentation	20	50%
Admission to High Dependency or Intensive Care Units	14	35%

Only 9 patients (22.5%) presented with the “classic triad” of fever, spinal pain and a neurological deficit. The range of days between presentation and suspected diagnosis in those without this classic triad of symptoms was significantly higher, between 0–30 days (median 1 day), compared to 0–3 days (median 0.5 days) in those who did.

The average C-reactive protein (CRP) on admission was 175.19 mg/L, ranging from 9–512 mg/L (median 138 mg/L). 6 (15%) had a CRP of less than 60 mg/L and 2 (5%) had a CRP less than 20 mg/L. On review of available results, none of the patients had a CRP within normal limits (8 results unavailable). 22 had positive blood cultures (55%) (10 unknown results). 15 had Staphylococcus aureus bacteraemia (68% of the positive results). Other blood cultures yielded growth of coagulase negative Staphylococcus (6 including Staphylococcus epidermidis (3) and Staphylococcus hominis (1)), Streptococcus agalactiae (2), Escherichia coli (1), Streptococcus constellatus (1), group B Streptococcus (1) and Enterococcus faecalis (1) (Some cultures grew more than one organism).

Excluding post-operative infections, the median interval between admission and commencing antibiotics was 2 days (0–33; SD 6.9). The median duration of antibiotics was 12 weeks, excluding mycobacterium infections. All patients had an MRI spine performed. The median number of days (for patients with native infections) between admission and the MRI being performed was 1 day (range 0–30 days (SD 6.8)). On average it took less than one day (0.85 days) for this to be reported.

The majority of infections affected the cervical and/or thoracic (n = 34, 85%) region. Multilevel involvement was evident in 23 patients (57.5%). Table 3 details levels affected.

**Table 3:** The spinal level affected.

Level of infection	Number
Thoracic	17 (42%)
Cervical	9 (22.5%)
Lumbar	4 (10%)
Cervical and Thoracic	2 (5%)
Cervical and Lumbar	2 (5%)
Cervical, thoracic and lumbar	2 (5%)
Thoracolumbar	2 (5%)
Unknown	1 (2.5%)
Lumbosacral	1 (2.5%)

29 patients underwent surgery and almost half of these (14) had instrumentation (48.3%). 7 required further surgery (4 with existing metalwork, 3 without; no bias towards those with metalwork was identified).

15 (37.5%) patients deteriorated neurologically following hospital admission. Of these, 10 had not had surgery before deterioration (5 managed conservatively throughout and 5 went on to have surgery after the deterioration). The remaining 25 patients (62.5%) presented with neurological deficits and did not experience further neurological deterioration. Of those who deteriorated neurologically, 8 were already on antibiotics (including one patient later diagnosed with mycobacterium tuberculosis associated spinal infection and one on antibiotics for a chest infection), and 5 were not. Pre-deterioration antibiotic status was not clear in 2 others.

No significant difference in time to suspected diagnosis (p = 0.55), time to antibiotics (p = 0.56) or time to MRI (p = 0.23) was demonstrated between those who did and did not deteriorate neurologically.

7 patients sustained complete spinal cord injuries (17.5%), 5 patients were AIS B (12.5%), 8 were AIS C (20%), and 19 were AIS D (47.5%). 1 had cauda equina syndrome. There was a notable absence of detailed AIS documentation during the acute treatment phase and often AIS examinations were delayed until transferred to the spinal injuries centre. Therefore, we are unable to develop conclusions regarding neurological changes.

All patients underwent an inpatient rehabilitation programme. On discharge, 24 patients (60%) required a self-propelling wheelchair to mobilise, 7 were walking with a frame, 5 were walking with one or two sticks/crutches, 3 required powered wheelchairs and 1 was bedbound (palliative). The majority of patients were independent for personal activities of daily living on discharge (23, 57.5%). 13 others required assistance and 4 were fully dependent.

Regarding bladder management, on discharge 12 patients voided with control, 10 had a suprapubic catheter, 9 performed intermittent self-catheterisation, 5 had an indwelling urethral catheter, one had a convene, one had a urostomy (pre-injury bladder management for previous bladder malignancy) and one (3%) was anuric with regular haemodialysis (1 dataset missing). On discharge, 20 patients managed their bowels using enemas or suppositories, 14 had evacuated with control, 4 required daily manual evacuations and 2 had stomas (one of whom had ileostomy for Crohns disease pre-admission).

Only 13 patients (32.5%) were discharged directly home self-caring. 8 patients (20%) required a care package on discharge and 3 were discharged to a care home (7.5%). 7 were repatriated/transferred to other hospitals (17.5%), 3 required interim care (7.5%) and 2 required intermediate care. One self-discharged and one declined care (2 others unknown).

At one year post discharge, 6 (15%) patients had died; their mean age at death was 75.8 years (range 65–87). Those patients who died within the first year had a high incidence of co-morbidities.

## Discussion

Misdiagnosis of spinal infection is common and has been reported in 50 to 75% of cases [7–9]. A systematic review by Suppiah et al. states the absence of early neurologic symptoms delays the diagnosis when clinicians are faced with spinal pain with constitutional symptoms [9]. Diagnostic delays can range from 2 to 12 weeks [10]. Delays in diagnosis appear to frequently occur pre-hospital. This could be attributed to a low index of suspicion in the community and the high prevalence of back pain seen in this setting. Half the patients in this study had previously sought medical attention for the same symptoms, indicating low awareness amongst clinicians. As only a small percentage of patients present with classic symptoms such as pyrexia, there needs to be a low threshold for considering the diagnosis. This point has also been emphasised by other authors [10].

CRP levels have been reported to be elevated in over 90% of cases and a persistently elevated CRP is highly correlated with the presence of infection [3]. This is in keeping with our findings from this series. We postulate the routine use of this marker when investigating back pain could help in identifying cases of spinal infection.

Although the “classic triad” of fever, spine pain and neurological deficit has been considered a hallmark of spinal epidural abscess, the majority of our cohort did not present in this way. Delays may result from over-reliance on this triad. One study found that assessment of risk factors was a more sensitive method of screening in emergency departments [11]. In our series, those who had the symptom triad were identified earlier than those without those symptoms.

The lumbar region is typically cited as the most common site for spinal infection [9, 12–14]. The majority of our patients – drawn from those requiring inpatient rehabilitation - had infections in the cervical or thoracic region. A possible explanation is that lower level injuries may have been managed in outpatient or community rehabilitation settings and would not have been referred to a tertiary spinal injuries centre. Therefore, this cohort would not have been highlighted in our search. An alternative explanation is that with lower level lesions, there is greater space in the canal and subsequently less risk of compression and consequent neurological deficit. In addition, the overall neurological deficit associated with lower lesions may have been less dense due to the greater space in this area with decreased risk of compression, and therefore may not have required referral to a specialist spinal injuries centre. A review by Karadimas EJ et al. corroborated this, observing that spinal infections were more common in the lumbar spine, but that neurological complications were commoner in thoracic and cervical infections [15]. A faster onset of neurological deficits in thoracic and cervical spine infection compared with lumbar lesions has been described [16].

A large proportion of our cohort deteriorated neurologically after diagnosis. 2/3 of these had not had surgery and several had been on antibiotics. It is difficult to pinpoint the exact cause of neurological deterioration amongst these patients. General causes for deterioration include embolism, compression, recurrence of an infective collection, ischaemia, and pathological fractures. Other studies have reported neurologic deterioration despite appropriate antibiotics [7, 17]. Therefore close neurological observation is indicated. The possibility of occult vertebral instability contributing to neurological deterioration has been suggested in conditions such as SCIWORA [18]. Whether such instability occurs with discitis is unclear. The described mechanisms associated with the development of neurological deficits in pyogenic vertebral osteomyelitis include epidural abscess in the narrowing canal, septic embolism, cord ischaemia and a compressive fracture [17].

There is no specific consensus on what constitutes diagnostic delay. Davis et al. [11], defined diagnostic delay as either multiple attendances at the emergency department before diagnosis, or admission without a diagnosis and over 24 h to a definitive study [11]. In their analysis, Davis et al. found that over 75% of patients experienced diagnostic delays. In this review, there were several patients who met the above criteria for a diagnostic delay.

The University of Michigan guidelines states that in cases of suspected spinal infection, immediate spine imaging should be performed, ideally within 6 h. If neurological deficits are present imaging should be performed within two hours and biopsy obtained within 24 h [5]. Prompt MRI imaging is necessary to avoid diagnostic delay [19]. We speculate reasons for delay to MRI could be due to the urgency of the diagnosis not being appreciated, lack of availability or access to MRI and delays in transfers between hospitals.

Whilst these patients have shown improvements following treatment and rehabilitation (the majority were discharged as being self-caring), life-changing disabilities persist. In our study 17.5% patients had complete neurological injuries and 67.5% were discharged as wheelchair users. This reiterates the significant morbidity associated with this condition, as well as the financial cost of life-long follow-up and care to the system and the individual.

Limitations of this study include the relatively small sample size, reflecting the uncommon nature of this condition. Additionally, some datasets were missing or unobtainable – the majority of patients were transferred from other hospitals where access to electronic records was not possible. There is also the inherent skew associated with analysing a cohort of patients who suffered the most severe consequences – we only included only persons who suffered a spinal cord injury and needed rehabilitation. Therefore the inferences made are not representative of the population at large.

We recommend:

1. Close neurological monitoring of those suspected or confirmed to have a diagnosis of spinal infection, whether they are on antibiotics or not.
2. Do not rely on the classic triad of symptoms.
3. Consideration of the use of inflammatory markers when investigating back pain.
4. Emphasise risk factors rather than symptoms alone to appropriately increase suspicion, with the common presenting complaint of spinal pain.

## Data Availability

The data that support the findings of this study are available from the corresponding author, [LG], upon reasonable request.
